# Reduced Cerebellar BDNF Availability Affects Postnatal Differentiation and Maturation of Granule Cells in a Mouse Model of Cholesterol Dyshomeostasis

**DOI:** 10.1007/s12035-023-03435-3

**Published:** 2023-06-14

**Authors:** Micaela Lucarelli, Serena Camuso, Chiara Di Pietro, Francesco Bruno, Piergiorgio La Rosa, Daniela Marazziti, Maria Teresa Fiorenza, Sonia Canterini

**Affiliations:** 1 Division of Neuroscience, Dept. of Psychology, University La Sapienza, Rome, Italy; 2 PhD Program in Behavioral Neuroscience, Sapienza University of Rome, Rome, Italy; 3 Institute of Biochemistry and Cell Biology, Italian National Research Council (CNR), I-00015 Monterotondo Scalo, Italy; 4 Regional Neurogenetic Centre (CRN), Department of Primary Care, ASP, 88046 Lamezia Terme, Catanzaro Italy; 5 Association for Neurogenetic Research (ARN), 88046 Lamezia Terme, Italy; 6 European Center for Brain Research, IRCCS Fondazione Santa Lucia, Rome, Italy

**Keywords:** Niemann-Pick type C1 disease, Npc1, Cerebellum, TrkB receptor, Cerebellar glomerulus

## Abstract

Niemann-Pick type C1 (NPC1) disease is a lysosomal lipid storage disorder due to mutations in the *NPC1* gene resulting in the accumulation of cholesterol within the endosomal/lysosomal compartments. The prominent feature of the disorder is the progressive Purkinje cell degeneration leading to ataxia.

In a mouse model of NPC1 disease, we have previously demonstrated that impaired Sonic hedgehog signaling causes defective proliferation of granule cells (GCs) and abnormal cerebellar morphogenesis. Studies conducted on cortical and hippocampal neurons indicate a functional interaction between Sonic hedgehog and brain-derived neurotrophic factor (BDNF) expression, leading us to hypothesize that BDNF signaling may be altered in *Npc1* mutant mice, contributing to the onset of cerebellar alterations present in NPC1 disease before the appearance of signs of ataxia.

We characterized the expression/localization patterns of the BDNF and its receptor, tropomyosin-related kinase B (TrkB), in the early postnatal and young adult cerebellum of the *Npc1*^*nmf164*^ mutant mouse strain.

In *Npc1*^*nmf164*^ mice, our results show (i) a reduced expression of cerebellar BDNF and pTrkB in the first 2 weeks postpartum, phases in which most GCs complete the proliferative/migrative program and begin differentiation; (ii) an altered subcellular localization of the pTrkB receptor in GCs, both in vivo and in vitro; (iii) reduced chemotactic response to BDNF in GCs cultured in vitro, associated with impaired internalization of the activated TrkB receptor; (iv) an overall increase in dendritic branching in mature GCs, resulting in impaired differentiation of the cerebellar glomeruli, the major synaptic complex between GCs and mossy fibers.

**Supplementary Information:**

The online version contains supplementary material available at 10.1007/s12035-023-03435-3.

## Introduction

Niemann-Pick type C1 disease (NPC1) is a rare autosomal recessive disorder caused by mutations in the *Npc1* gene, which encodes a transmembrane protein that mediates the egress of low-density lipoprotein (LDL)–derived cholesterol from the endosomal/lysosomal compartments [1, 2]. Despite heterogeneous symptomatology, reviewed in [2], the prominent feature of NPC1 is the progressive degeneration of the cerebellar Purkinje cells (PCs), leading to heterogeneous neurological manifestations such as cognitive impairment, vertical supranuclear saccade, clumsiness, gait disturbance, neurodegeneration, and cerebellar ataxia [3–7].

Some evidence suggests that abnormal cholesterol metabolism and endosomal/lysosomal alterations, associated with *Npc1* deficiency, affect neurotrophic signaling: *Npc1* KO mice and PC12 cells treated with U18666A, a well-known inducer of the NPC1 phenotype [8], exhibit an upregulation of nerve growth factor (NGF) signaling along with prominent endocytic dysfunction, including an increased size of tropomyosin receptor kinase A (TrkA)–containing endosomes and reduced recycling of this receptor [9].

Our previous studies highlighted early defects in cerebellar development that precede neurodegeneration in adults. In particular, in *Npc1* mutant mice, we demonstrated that a defective proliferation of cerebellar granule cells (GCs) is associated with reduced availability and defective reception of Sonic hedgehog (Shh) signal at the primary cilium [10–12] and with a delay in the acquisition of complex motor skills [13]. Shh is known to be a critical factor regulating the BDNF expression and signaling [14, 15], stimulating the expression and secretion of this neurotrophic factor, through post-translational mechanisms and the activation of the subsequent signaling pathways of the tropomyosin kinase receptor B (TrkB) [16].

Moreover, cultures of embryonic striatal neurons isolated from *Npc1* mutant mice show reduced signaling of BDNF, via the loss of activation of its high-affinity TrkB receptor [17], while the BDNF and TrkB null mice show severe phenotype and exhibit ataxia [18–22] and the selective reduction of cerebellar BDNF expression has been described in ataxic stargazer and waggler mice [21, 23, 24].

The BDNF pathway represents a key determinant of cerebellar development and function and is highly expressed in GCs and deep cerebellar nuclei, while its receptor is mainly expressed in PCs, GCs, interneurons, and glia [20, 25–27]. Interestingly, this neurotrophin can act as both an autocrine and a paracrine factor. The TrkB activation induces an autocrine release of BDNF from GCs in the internal granule layer (IGL), which maintains the BDNF gradient itself, whereas BDNF released from GCs also controls PC differentiation in a paracrine manner [26–28].

In this study, we hypothesized that BDNF-TrkB signaling is impaired in the cerebellum of *Npc1* mutant mice and that, together with the previously observed Shh reduction, this anomaly contributes substantially to the postnatal cerebellar abnormalities already demonstrated in *Npc1*-deficient mouse models. To this purpose, we exploited the hypomorphic mouse model *Npc1*^*nmf164/ nmf164*^ (*Npc1*^*nmf164*^) which, in contrast to the classical knockout model, carries a point mutation in a region of the *Npc1* gene where a high percentage of mutations are found in our species [29].

The evidence we provide in this study shows an altered expression, localization of the BDNF signaling in the early stages of postnatal cerebellar development. Furthermore, in GCs isolated from mutant mice, we demonstrated a reduced responsiveness to exogenous BDNF and an altered program differentiation of these cells.

Overall, our findings support the evidence by which the early dysregulation of the BDNF signal is an important alteration of postnatal developmental of *Npc1*^*nmf164*^ mice, contributing to the establishment of morpho-functional abnormalities in the cerebellar cortex, also present during the pre-symptomatic stages of the disease.

We believe that a better understanding of the additional physiological roles played by BDNF in a mouse model of lipid dyshomeostasis, such as *Npc1*^*nmf164*^, could help to elucidate the link between neurotrophin, cholesterol metabolism, and brain pathology.

## Materials and Methods

### Animals

*Npc1* ^*nmf164*^ mice with a BALB/cJ background, carrying a single point mutation (D1005G) in the *Npc1* gene [29], obtained by mating heterozygous animals were used in this study. Genotypes were identified by PCR followed by restriction enzyme digestion [29]. Animals were maintained in our animal facility in accordance with Sapienza University guidelines and exposed to a 12-h light-dark cycle, receiving food and drinking water ad libitum*.* Experimental determination: animal experimental protocols and related procedures were approved by the Italian Ministry of Public Health. All efforts were made to minimize animal suffering, according to Italian Legislative Decree 26/2014 and European Directive 2010/63/EU.

### RNA Preparation and Real-time RT-PCR

Total RNA was extracted from postnatal day (PN) 4, 8, 11, 15, and 30 *Npc1*^*nmf164*^ and wild-type (*wt*) littermate cerebella using the RNeasy mini kit (Qiagen, Hilden, Germany) [11]. Custom-designed primer pairs specific for BDNF were 5′-CATAAGGACGCGGACTTGTACA-3′ and 5′-AGACATGTTTGCGGCATCCA-3′. Relative gene expression levels were analyzed using the 2^-Delta Delta Ct method (∆∆Ct) [30], normalized to the expression of S16 ribosomal RNA (*rps16*) as housekeeping gene (5′AGGAGCGATTTGCTGGTGTGG-3′ and 5′-GCTACCAGGGCCTTTGAGATGGA-3′). The expression levels were also normalized to *Gapdh* (not shown) observing similar results. Statistical significance was determined with unpaired Student’s *t* test.

### Primary In Vitro Culture of Cerebellar GCs and Chemotaxis Assay

In vitro primary GC cultures were prepared from cerebella of PN6–PN7 *wt* and *Npc1*^*nmf16*4^ littermates, as previously described [31].

A Boyden chamber cell migration assay was performed as described in [32]. Briefly, purified GCs in serum-free Dulbecco’s Modified Eagle Medium (DMEM) were placed in the poly-lysine precoated upper Boyden chamber while BDNF (40 ng/mL, #B-250, Alomone Labs, Jerusalem, Israel) was added to the lower chambers in the same medium. After overnight incubation at 37°C in 5% CO2, the upper surface of the membranes was scraped free of cells and debris. The membranes and covers were washed, fixed, and stained with Hoechst (Hoechst-33258, Invitrogen, Milan, Italy). Cells adhering to the underside of the membrane were counted under an epifluorescence Zeiss microscope with a 10× objective. The total number of cells that had migrated was calculated in five adjacent fields and the average values of each experimental group were expressed in arbitrary units, normalizing to the untreated control.

### Protein Extract Preparation and Western Blot Analysis

Total protein from cerebella of PN4, PN8, PN11, PN15, and PN30 *Npc1*^*nmf164*^ and *wt* littermates or from purified GCs (DIV0) was extracted with RIPA Buffer (Sigma-Aldrich, Milan, Italy) and processed using western blot analysis, as described in [33]. Primary antibodies used were BDNF (Santa Cruz Biotechnology, #sc-546, Milan, Italy), TrkB (Sigma Aldrich, #ZRB1281, Milan, Italy), pTrkB (Sigma Aldrich, #SAB4503785, Milan, Italy), and EEA1 and β-actin (GeneTex, #GTX634169, North America, Elabscience, #AF0342, USA). Secondary horseradish peroxidase–conjugated secondary antibodies used were mouse IgG (Cell Signaling, #7076S, Milan, Italy), mouse IgM (Santa Cruz Biotechnology, #sc-2064, Milan, Italy), and rabbit IgG (Vector Laboratories, #PK-6101, Burlingame, CA, USA). The intensity of each band was normalized to the β-actin signal intensity and the average values were expressed in arbitrary units, as a ratio to the *wt* mean values (ChemiDoc XRS Imager, Bio-Rad).

Early endosomes were isolated from cerebella of PN8 *wt* and *Npc1*^*nmf164*^ mice using Trident Endosome Isolation Kit (GeneTex, #GTX35192, North America), according to the manufacturer’s instruction. Pellets containing purified early endosomes were resuspended into Minute^TM^ Denaturing Protein Solubilization Reagent (Invent Biotechnologies Inc, #WA-009, Plymouth, USA) and protein concentration was determined by the Bradford assay (Thermo Fisher Scientific)

### Immunohistochemistry and Immunofluorescence Analysis

To collect brains, pups were killed by decapitation, whereas adult mice were deeply anesthetized via the intraperitoneal injection of xylazine (20mg/Kg) and ketamine (34mg/kg) and transcranially perfused with 4% PFA in 0.1M PBS. Upon isolation, brains were post-fixed in 4% PFA overnight at 4°C and sagittally sectioned with a vibratome (S1000, Leica, Milan, Italy) or embedded in paraffin (Paraplast Tissue Embedding Medium, Leica Biosystem, Milan, Italy) and sectioned with a microtome (Microm hm330, Leica, Milan Italy). For BDNF and synaptophysin (Syp) immunohistochemistry, free-floating vibratome sections or rehydrated cryosections were incubated in hydrogen peroxide for 15 min to inactive the endogenous peroxidase, rinsed 3 times for 5 min in Tris-Buffered Saline (TBS), and permeabilized with 0.3% Triton X-100 (Sigma-Aldrich, Milan, Italy) in TBS for 15 min. After 3 washes in TBS, sections were incubated in blocking solution containing 10% rabbit or goat serum, 1% Bovine Serum Albumin (BSA), and 0.2% Triton X-100 for 1 h at room temperature (RT) and then incubated with rabbit polyclonal antibodies directed to BDNF (Santa Cruz Biotechnology, #sc-546, Milan, Italy) or goat polyclonal antibodies directed to synaptophysin (Santa Cruz Biotechnology, #sc-7568, Milan, Italy) overnight at 4°C. After 3 washes in TBS, immunoperoxidase staining was visualized using the ABC Elite Kit with 3,3′-diaminobenzidine (DAB) as chromogen (Vector Laboratories, Burlingame, CA, USA), according to the manufacturer’s protocol. Nuclear counterstaining was performed by incubating sections with 0.5% methyl green solution (0.5% methyl green, 0.1M sodium acetate buffer in water; Cat. # 42590, Sigma Aldrich, Milan, Italy) for 5 min at 60°C. After dehydration and clarification, sections were cover-slipped with Vectamount (Vector Laboratories, Burlingame, CA, USA). To quantify BDNF expression, digital images of IHC-labeled sections, acquired by the epifluorescence Zeiss microscope with a 10× objective, were analyzed with ImageJ Fiji software (National Institutes of Health) as previously described in [34].

The quantification of cerebellar glomeruli was performed by outlining manually synaptophysin-positive cell/area (representing mossy fiber endings at the levels of glomeruli), by using “freehand contour drawing” from the Neurolucida Software (version 8.0 by MBF Bioscience, Inc.), connected to an Olympus BX53 microscope with 40× and 100× immersion objective lens. The depth of the z-plane was varied to ensure optimal clarity for an accurate and precise morphological 3D reconstruction. The number, area (total number of microns present in a single object, μm2), and tortuosity (the ratio of the Euclidian distance between two endpoints and the axonal path length between those points) of glomeruli were determined at PN11 and PN30 in *wt* and *Npc1* mutant mice.

To further study the cerebellar cytoarchitecture complexity, we also performed a Sholl analysis of 3D reconstructed GCs and glomeruli by analyzing the number, diameter, and distance between the GCs as well as between the GCs and mossy fiber (MF) endings. Obtained data were statistically evaluated with Student’s *t* test.

For immunofluorescence (IF) analysis, cerebellar slices and in vitro differentiated GCs (DIV3) were fixed with 4% PFA and incubated for 1 h at RT with Alexa Fluor 488– or 555–labeled secondary antibodies (Jackson ImmunoResearch Laboratories, Milan, Italy) while the nuclei were stained with 4′,6-diamidino-2-phenylindole (DAPI) in PBS [35]. Immunofluorescence was routinely detected with a TCS SP5 laser scanning confocal microscope (Leica Microsystems, Mannheim, Germany). Semi-quantitative analysis of BDNF immunoreactivity was performed using the software ImageJ Fiji.

Subcellular localization of pTrkB and early endosome antigen 1 (EEA1) (Gene Tex, #GT1081, Prodotti Gianni, Milan, Italy) by immunofluorescence (IF) analysis was performed on GCs after BDNF stimulation, using a commercially available chemotaxis chamber, with three observation areas (2×1mm^2^) (μ-Slide Chemotaxis, ibiTreat, #80326, ibidi GmbH, Grafelfing, Germany). Briefly, GC suspension (1×10^6^cells/mL) was placed in the central observation area, whereas serum-free DMEM medium and attractant medium (40 ng/mL of BDNF in serum-free DMEM) on the lateral reservoirs. After incubation at 37°C in 5% CO_2_ for 2 days, cells were fixed in 4% paraformaldehyde (PFA), washed 3 times in phosphate-buffered saline (PBS), and immunostained as described below.

### Golgi Staining and Morphological Analysis of Cerebellar GCs

PN30 *Npc1*^*nmf164*^ and *wt* brains were processed using the Golgi-Cox protocol as previously described [36]. First, brains were transferred into a Golgi-Cox solution for 7 days and then for 1 week at 4°C in a cryoprotectant solution consisting of 30% sucrose in PBS. Brains were cut with a Vibratome (S1000, Leica, Milan, Italy), obtaining sagittal sections (200 μm), which were placed on gelatin-coated slides, dehydrated, developed with a 3:1 ammonia solution and 5% sodium thiosulfate, and finally rehydrated and mounted. GCs were easily recognized due to their characteristic morphology, featuring a very small soma and short dendrites that terminate in claw-shaped endings. Neuronal soma and dendritic branching were manually traced with Neurolucida Software with a 100× immersion objective lens. Only neurons that exhibited complete Golgi impregnation and intact dendrites were selected for reconstruction. A sholl analysis has been used to analyze morphometry and dendritic branching of Golgi-Cox impregnated neurons. Regarding the morphology and dendritic branching of GCs, the following parameters were analyzed: soma perimeter (μm) and soma area (μm^2^); the number of dendrites, nodes, and ends. Furthermore, a total dendritic analysis was performed considering the following components: total dendritic length (μm), total dendritic surface (μm^2^), total dendritic volume (μm^3^), and mean dendritic diameter (μm).

### Statistics

All the statistical analyses were performed with GraphPad Prism 8.0 (GraphPad Software, Inc., San Diego, CA). Quantitative data were analyzed by Student’s *t* test. Differences between groups were considered significant at a *p* value of <0.05 and expressed as mean ± S.D.

## Results

**Fig. 1:**
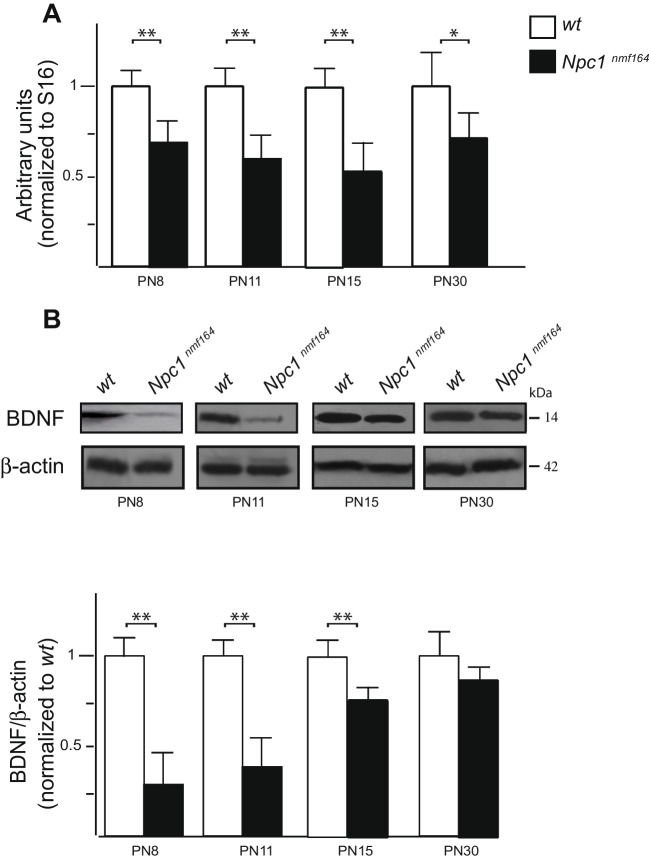
*Npc1*^*nmf164*^ mice display a reduced BDNF expression in the postnatal developing cerebellum. Relative quantification of BDNF mRNA (**A**) and representative immunoblots of BDNF protein expression levels (**B**) in *wt* (empty bars) and *Npc1*^*nmf164*^ (full bars) mice at increasing time points. The BDNF transcript and protein levels were normalized to the expression levels of the housekeeping gene S16 (unpaired *t* test * *p* < .05 versus *wt*. ** *p* < .01 versus *wt*) and β-actin (unpaired *t* test **p < .01), respectively. Histograms indicate the abundance expressed as mean ± SD (5 mice/group).

To gain insights into BDNF expression in the developing cerebellum of *Npc1*^*nmf164*^ mice, BDNF transcript and protein levels were determined at PN8, PN11, PN15, and PN30 (Fig. 1A, B). A significant reduction of both transcript and protein levels was observed in *Npc1*^*nmf164*^ mice compared to *wt*, starting from PN8, with no significant difference found at an earlier time point—PN4 (Figure 1 SM). This BDNF expression reduction persisted up to PN15, whereas at PN30, despite BDNF transcript levels still significantly reduced (Fig. 1A), the expression of the BDNF protein appeared not to vary between *wt* and *Npc1*^*nmf164*^ mice (Fig. 1B).

**Fig. 2:**
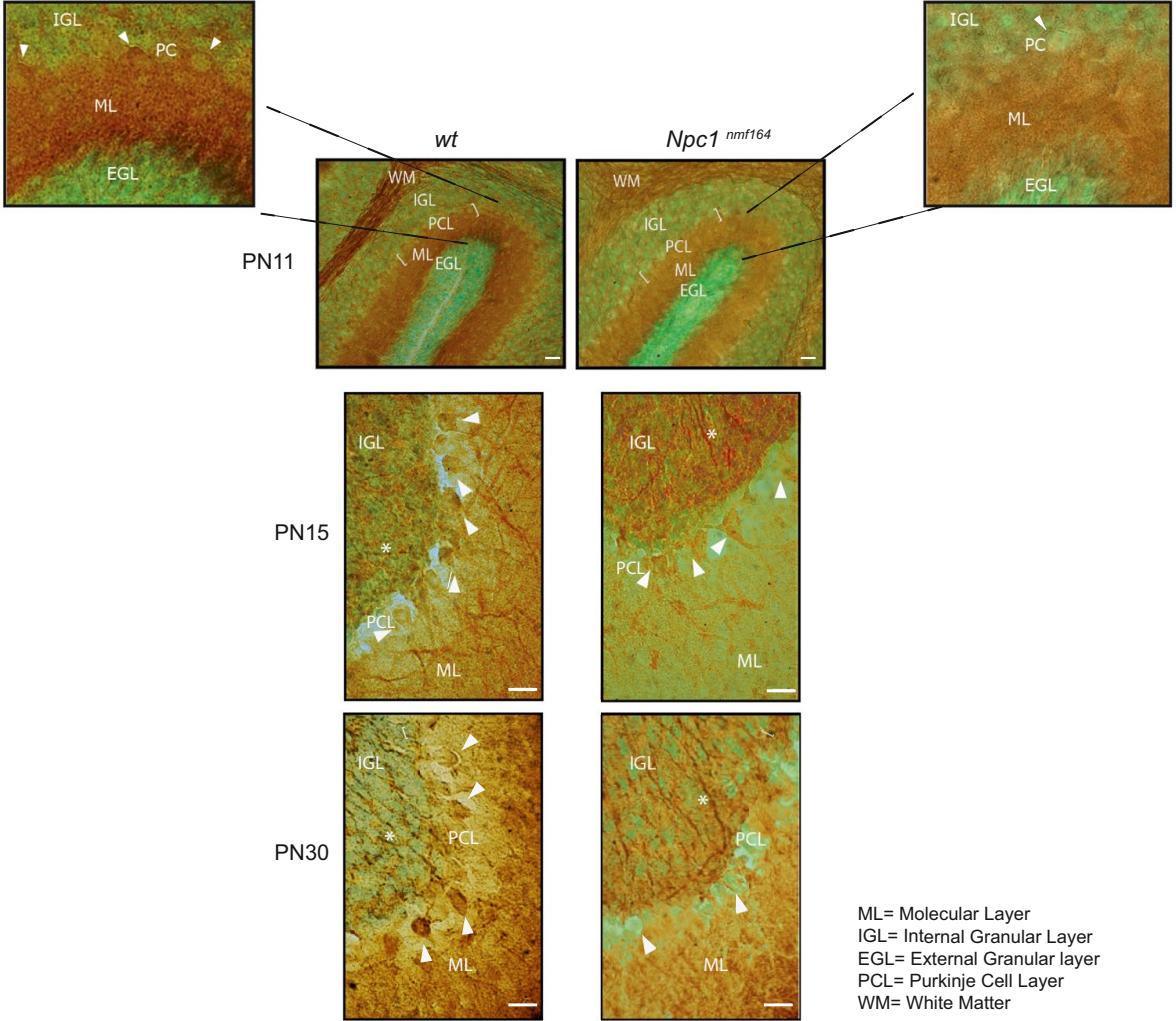
*Npc1*^*nmf164*^ mice display abnormal BDNF protein localization along cerebellar cortex development. Representative fields of parasagittal sections of *wt* and *Npc1*^*nmf164*^ mouse cerebella are shown in the figure. Sections of PN11, 15, and 30 of *wt* and *Npc1*^*nmf164*^ mice (3–4 mice/group; 3–4 sections/mouse) were immunostained with anti-BDNF antibody (brown) and nuclei counterstained with methyl green. Higher magnifications of the PN11 fields are shown in upper panels. EGL, external granular layer; ML, molecular layer; PCL, Purkinje cell layer; IGL, internal granular layer; asterisks point mossy fibers; white arrowheads point PCs. scale bars: 40 μm

To detect and localize BDNF protein in the developing and adult cerebellum (PN11, 15, 30), we carried out immunohistochemical analyses (Fig. 2) associated with a semi-quantitative determination of BDNF expression on parasagittal brain slices of *wt* and mutant mice (Table 1 SM).

The typical BDNF expression in the cerebellar cortex, characterized by a greater BDNF protein in the internal granular layer (IGL) and molecular layer (ML) compared to the external granular layer (EGL) [20, 28, 37], was evident in both *wt* and *Npc1* mice. However, in the *Npc1*^*nmf164*^ mice, the overall intensity of the BDNF expression was reduced, as shown in Fig. 2. In PN11 *Npc1* mutant mice, indeed, BDNF protein expression was barely detected in the EGL and poorer in the ML and IGL when compared with the *wt* littermates. In detail, strong immunoreactivity was observed in the PCs body and white matter (WM) fiber tracts in the *wt* mice in contrast to the lower BDNF immunoreactivity detected in the *Npc1*^*nmf164*^ mice (Fig. 2). Similar results were observed at PN8 (data not shown).

A few days after, at PN15, when GC proliferation in the EGL is almost completed [38], the BDNF immunoreactivity was widely distributed in the cerebellar cortex layers of *wt* mice [39], with a strong signal in the mossy fibers (MFs) (as observed using cadherin as cerebellar MF marker, data not shown), representing, beside the GCs, the most important source of BDNF in the cerebellum [25, 40] (Fig. 2). In addition, a slight BDNF signal was observed in the ML, soma (arrowhead), and dendritic tree of PCs. In *Npc1* mutant mice, overall BDNF expression throughout the cerebellar cortex was reduced, with the MF terminals showing a subtle alteration in their morphology compared to *wt* ones (Fig. 2).

Finally, at PN30, cerebellar expression of BDNF was clearly detected in the PCs (arrowhead) and IGL of *wt* mice. Interestingly, in *Npc1*^*nmf164*^ mice, BDNF signal was completely absent in PCs, while strong immunostaining was observed in MFs, showing an oversized and hypertrophic morphology in comparison to *wt* (Fig. 2 and Figure 2 SM).

### In Vivo and In Vitro Cerebellar Characterization of pTrkB Receptor Expression in Npc1^nmf164^ Mice

**Fig. 3:**
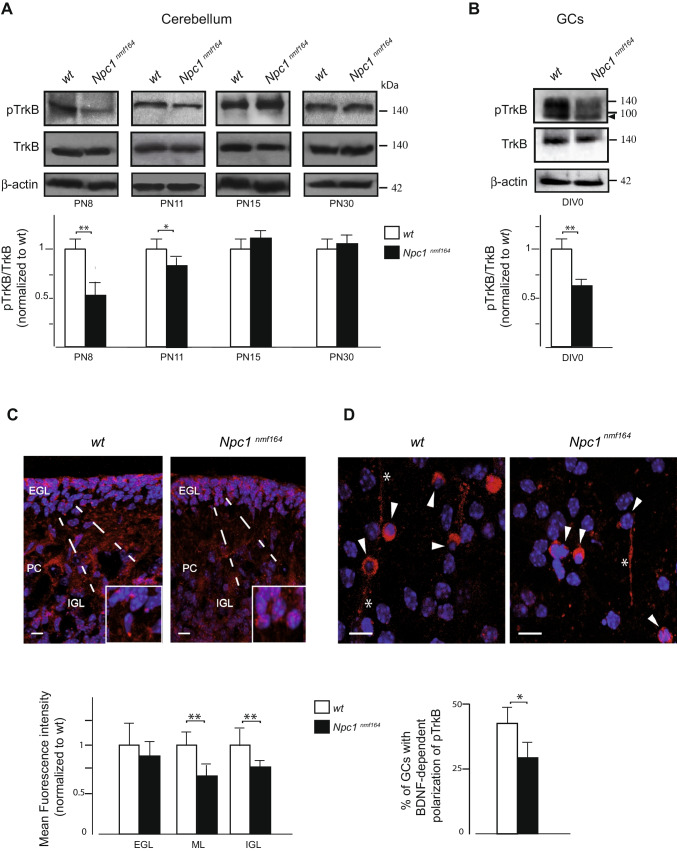
Abnormal pTrkB receptor expression and localization are observed in the early stage of cerebellar development and in vitro GCs of *Npc1*^*nmf164*^ mice. **A**, **B** Representative immunoblots of pTrkB protein expression in extracts from *wt* and *Npc1*^*nmf164*^ mice at different stages of cerebellar development (PN8,11, 15, and 30) and in DIV0 GCs. Western blot quantification of pTrkB in *wt* (white bars) and *Npc1*^*nmf164*^ (black bars) mice. Histograms indicate the abundance (mean ± SD) of each protein determined by the densitometry of protein bands obtained by taking total TrkB (TrkB) as an internal reference. Arrowhead indicates TrkB truncated form present in GCs. Significance is shown as a *p* value calculated using an unpaired *t* test. *p < .05 versus *wt.* **p < .01 versus *wt* (*n* = 5 mice/group). **C**, **D** Representative fields of confocal images are shown in the figure. Detection of pTrkB receptor (red) and nuclei (Hoechst 33258, blue) in PN8 cerebellar sections and in DIV3 GCs. The GCs in the EGL are magnified in the boxed regions of panel C. Scale bar: 20 μm. Comparison of the mean fluorescence intensity (y-axis) generated by staining sagittal sections of PN8 cerebellar cortex layers of *wt* and *Npc1*^*nmf164*^ mice with antibodies against pTrkB receptor. **D** GCs expressing pTrkB receptors in the soma (arrowheads) and in the leading process (asterisks) are indicated. Data shown represent the means ± SD and were evaluated by unpaired *t* test. **p < .01 versus *wt* (*n* = 3 mice/group). ML, molecular layer; PC, Purkinje cell; IGL, internal granular layer. Quantitative analysis of in vitro GCs with BDNF-dependent pTrkB polarization in *wt* and *Npc1* mutant mice is shown as a *p* value calculated using an unpaired *t* test. *p < .05 versus *wt*

To gain insight into signaling and reception of cerebellar BDNF, levels of the activated phosphorylated form of the TrkB receptor (pTrkB), relative to total TrkB (TrkB), were assessed in cerebellar extracts of PN8, PN11, PN15, and PN30 *wt* and *Npc1*^*nmf164*^ mice (Fig. 3A). A significant reduction of pTrKB was observed only at PN8 and PN11 in *Npc1*^*nmf164*^ mice compared to controls (Fig. 3A). This finding is confirmed also in purified GCs from mutant mice (Fig. 3B) and by immunofluorescence of PN8 cerebellar sections that showed lower pTrkB receptor expression, especially at the level of ML and IGL in *Npc1*^*nmf164*^ compared to *wt* (Fig. 3C).

Since it is difficult to determine the intracellular localization of pTrkB in cerebellar slices, due to the fact that TrkB-expressing neurons are densely packed in the EGL and IGL layers, we exploited in vitro cultures of cerebellar GCs to investigate the subcellular localization of TrkB upon BDNF stimulation. GCs purified from neonatal *wt* and *Npc1* mice were placed in ibidi μ slide and BDNF stimulus (40 ng/mL) was added in serum-free DMEM only in one of the two chambers, thus generating a gradient of BDNF. After 72 h, the immunofluorescence analysis was performed, observing that a smaller fraction of GCs isolated from *Npc1* mice had the typical polarized accumulation of the pTrkB receptor (red) in the soma (arrowheads) and in the leading stimulus-oriented processes (asterisks) (Fig. 3D), which are required to direct GC cell migration and differentiation in the IGL [22]. In contrast to the polarized localization of pTrkB puncta observed in *wt* GCs, pTrkB immunostaining in *Npc1*^*nmf164*^ GCs exhibits a more homogeneous distribution pattern in the soma (arrowheads) and a significantly reduced fraction of cells with BDNF-dependent polarization of pTrkB (Fig. 3D), like that seen in vivo (Fig. 3C). Significantly, after culturing for 7 days (DIV7), GCs obtained from neonatal *Npc1* mice did not exhibit any intracellular cholesterol accumulation, as observed through Filipin staining (Figure 3 SM).

**Fig. 4:**
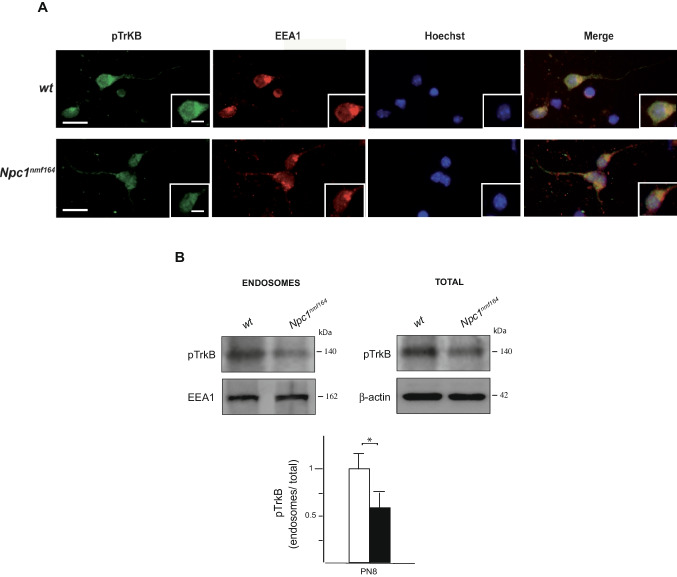
Reduced pTrkB localization in early endosomes of *Npc1*^*nmf164*^ cerebella. **A** BDNF-dependent polarization and colocalization of pTrkB and early endosome antigen 1 (EEA1) were visualized by immunostaining with anti-pTrkB (green) and anti-EEA1 (red), respectively, in BDNF stimulated DIV3 GCs of *wt* and *Npc1*^*nmf164*^ mice. Scale bar: 20 μm; 10 μm. **B** Representative immunoblots of pTrkB protein expression in cerebellar extracts from *wt* and *Npc1*^*nmf164*^ mice at PN8. Western blot quantification of pTrkB and EEA1 in *wt* (white bars) and *Npc1*^*nmf164*^ (black bars) mice. Histograms indicate the abundance (mean ± SD) of each protein determined by the densitometry of protein bands obtained taking EEA1, for endosomal fraction, and β-actin, for total homogenate, as internal reference. Significance is shown as a *p* value calculated using an unpaired *t* test. *p < .05 versus *wt* (*n* = 3 mice/group)

To gain a deeper insight into the interplay between pTrkB subcellular localization and GCs response to BDNF, we determined the fraction of pTrkB localizing to the endosomal compartment. Indeed, previous studies have shown that pTrkB endocytosis in early endosomes, present in major processes of granule neurons, is a key developmental mechanism, which provides precursors with the polarization required for migration through BDNF gradient [22]. While the pTrkB receptor was found to localize in the early endosomes of leading processes of *wt* GCs exposed to BDNF gradient, as indicated by co-immunolocalization and western blot analysis using antibody against anti-early endosome antigen 1 (EEA1), the fraction of pTrkB co-localizing with EEA1 appeared significantly reduced in in vitro cultured GCs and in cerebellar extracts from mutant mice, as determined by immunofluorescence analysis of isolated GCs and western blot analysis of endosomal/total protein extracts, respectively (Fig. 4A, B). Such reduction corresponded to roughly 40% (Fig. 4B).

### Reduced Sensitivity to Exogenous BDNF of Primary Culture of Npc1^nmf164^ GCs

It is well established that, during cerebellar development, BDNF acts as a mitogenic and chemotactic factor, stimulating cerebellar granule cell precursors (GCPs) to migrate radially along Bergmann glia fiber, from EGL to IGL [28, 41]. To investigate whether the intrinsic responsiveness of GCs to the chemotactic effect of BDNF was altered in *Npc1*-deficient GCs, we performed a chemotaxis assay using isolated GCs.

**Fig. 5:**
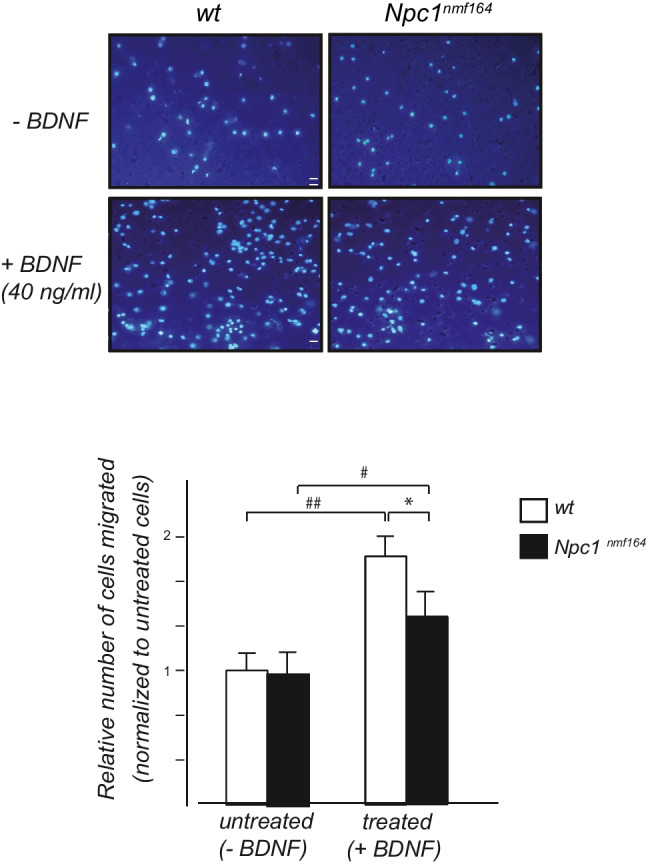
In the *Npc1* mutant mice, in vitro cultured GCs display a reduced BDNF chemotaxis response. Purified *wt* and *Npc1*^*nmf164*^ GCs were cultured for 24 h in Boyden chambers without (−BDNF) or with (+BDNF) 40 ng/mL BDNF in the lower chambers. GCs that migrated through the porous membrane into the lower chamber were directly quantified by fluorescence microscopy. Representative fields of migrated GCs, with nuclei staining with 0.5 μg/mL Hoechst-33258. Scale bar 20 μm. The results from five separate experiments, expressed as number of migrated cells normalized to untreated cells, are shown in the histograms. Treated *Npc1*^*nmf164*^ GCs (black bars) display a significant reduced chemotaxis response to BDNF compared to the treated *wt* cells (white bars). *p< .05 versus *wt* (*n* = 5 mice/group)

To this end, we used a Boyden chamber assay, wherein *Npc1*^*nmf164*^ and *wt* GCs isolated from PN6–7 cerebella were plated on one side of the porous membrane while in the lower chamber were added plain culture medium or BDNF- supplemented medium (40 ng/mL). In vitro chemotactic effects of BDNF were evaluated after 24 h by quantifying the cells that had migrated and adhered to the bottom side of the porous membrane. The nuclei of attached cells were visualized using Hoechst staining. In the absence of exogenous BDNF (−BDNF), we observed that the baseline motility of GCs did not change between the two experimental groups. In the presence of BDNF (+BDNF), however, only *wt* mice showed an approximately 2-fold increase in cell migration rate compared with control cells in a plain medium, as expected [22]. In contrast, GCs isolated from *Npc1* mutant mice were found to be less sensitive to the classical chemoattractant role of BDNF, resulting in a 45% decrease in cell migration rate compared to *wt* cells (Fig. 5).

### Changes in Dendritic Branching Are Present in the GCs of Adult Npc1^nmf164^ Mice

In addition to GC migration, BDNF-TrkB signaling also contributes to the later stages of GCs maturation in the developing cerebellum, in terms of differentiation of GCs into IGL and subsequent involvement in synaptic plasticity [42–47].

To highlight if and how the BDNF anomalies observed in *Npc1*^*nmf164*^ mice at earlier stages of cerebellar development were associated with alterations in GC differentiation program at later stages, we performed a Golgi staining of cerebellar sections obtained from PN30 mice, when the differentiation of these cells is completed in the IGL (Figs. 1–2).

Sholl analysis was performed to analyze neuronal morphometry and dendritic branching of Golgi-Cox impregnated neurons. In particular, the following parameters were analyzed: GC soma area, perimeter, the number of dendrites, nodes, and ends. Moreover, dendritic length, surface, and volume and mean dendritic diameter were measured.

**Fig. 6:**
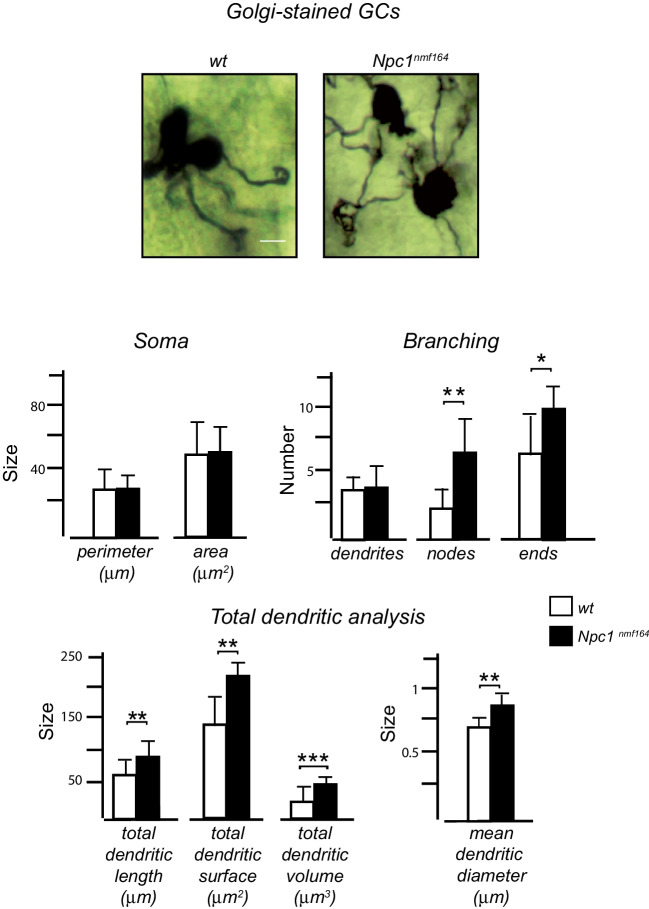
GCs of *Npc1*^*nmf164*^ mice exhibit abnormal dendritic morphology in the IGL layer. Representative fields of Golgi-impregnated GCs from the PN30 IGL of *wt* (left) and *Npc1*^*nmf164*^ (right) mice. (Scale bar: 10μm). Soma and dendrites of Golgi-stained neurons were traced, and morphological parameters were analyzed using Neurolucida Software. Unpaired Student’s *t* test analysis of soma (top left); number of dendrites, nodes, and ends (top right); total dendritic length, surface, and, volume (bottom left); and average diameter (bottom right) of cerebellar GCs were represented by histograms. Data are shown as mean ± SD; *p < .05; **p < .01; ***p < .001. (*n* cell for each genotype = 10). *wt* = white bars; *Npc1*^*nmf164*^ = black bars

While no difference in soma area and perimeter was observed between groups, and also the total number of dendrites did not vary between *wt* and *Npc1* mice, subtle anomalies of dendrite morphology were observed. In particular, Golgi-stained neurons displayed a significant increase in the number of nodes and dendritic endpoints, and in the total dendritic analysis parameters analyzed, as length, surface, volume, and diameter, in the *Npc1*^*nmf164*^ mice compared to the controls (Fig. 6).

### Synaptic Connectivity at Glomerular Rosette Is Altered in Npc1^nmf164^ Mice

Cerebellar GCs complete their differentiation in the IGL and connect, via their dendrites, with the MF axons, establishing synaptic contacts at the level of glomeruli, which are anatomically recognizable as “rosettes” [48–52] (Figure 4 SM). To gain an insight into the cytoarchitecture of the IGL, at PN11 and PN30, we have identified glomeruli by synaptophysin immunoreactivity, which represents a valid marker for glomeruli [53] and determined the number of GCs. As for glomeruli, we have investigated their density and shape, with reference to the overall area. To evaluate the complexity of the glomeruli, we analyzed the tortuosity and convolution of the positive synaptophysin area, considering that it is known that simple and less convoluted MF terminations are characteristic of immature glomerular rosettes [54, 55].

Furthermore, since rosette maturation is regulated both by secreted GC factors acting on MFs and by the number of contacts between GCs and MFs [25], we also estimated the number of GCs and studied how GCs arranged with each other and with respect to MFs.

**Fig. 7:**
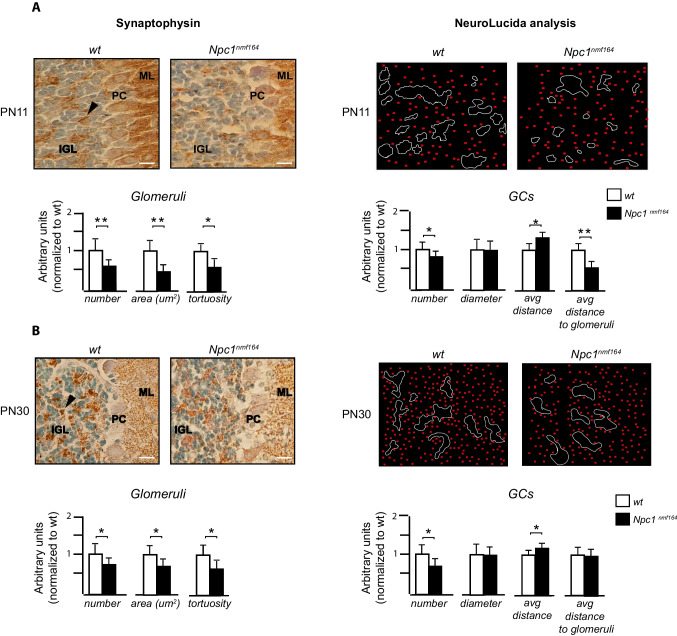
Morphological defects of cerebellar MF terminals maturation in the IGL of *Npc1*^*nmf164*^ *mice*. Synaptophysin (Syp) immunohistochemistry of cerebellar sagittal sections from three independently bred pairs of PN11 and PN30 *wt* and *Npc1* mutant mice, counterstained with methyl green (scale bar: 20 μm). The number of cells and morphological parameters (area and tortuosity) were analyzed with the Neurolucida Software. Moreover, the number of GCs and the distance between the GCs and among the GCs-glomeruli rosette were also analyzed in the same fields. The *Npc1*^*nmf164*^ mice exhibit fewer, smaller, and less complex Syp-positive area at the levels of glomeruli rosette in both stages analyzed, which was associated with a reduced number of GCs. Histograms represent statistical analysis with unpaired Student’s *t* test. Data are shown as mean ± SD *p < .05; **p < .01. *wt* = white bars; *Npc1*^*nmf164*^ = black bars. Arrowheads = MF terminals, ML, molecular layer; PC, Purkinje cell; IGL, internal granular layer

As shown in Fig. 7, PN11 *Npc1* mutant mice displayed a significant reduction in glomeruli number, area, and tortuosity when compared to the *wt* mice. Furthermore, although a greater distance between the GCs was found in the mutant mice due to a reduction in cell number [10], a significant decrease in the GC-to-glomerulus distance was also observed.

Similar results were observed at PN30, according to the stunted MF morphology observed in Fig. 2. However, although the GCs appear more spaced in the mutant mice, no difference was found in the distance among GCs and positive synaptophysin area between the two groups (Fig. 7).

## Discussion and Conclusion

This study provides strong evidence that *Npc1* mutant mice display dysregulation in the expression and localization of BDNF and its receptor during early cerebellar postnatal development associated with abnormalities in the differentiation and synaptic connectivity of the GCs. In detail, we found that in *Npc1*^*nmf164*^ mice (i) the expression of the mature form of BDNF is reduced in the first 2 postnatal weeks; (ii) the expression and subcellular localization of the pTrkB receptor is altered in the GCs; (iii) the differentiation program of GCs is compromised; and (iv) the maturation of MF-GC synapses is impaired.

The results of this study support and expand recent findings that sustain the loss of Purkinje cells as an outcome of a different impairment of neurodevelopment in NPCD disease. Our previous studies have demonstrated that the generation and reception of the Shh signal at the primary cilium are negatively affected by *Npc1* deficiency [11], in combination with defective GC proliferation in *Npc1* mutant mice [10].

Although Shh signaling plays a key role during the pre- and postnatal stages of cerebellar development, regulating events such as foliation and patterning, other molecules, like BDNF, fine-tune postnatal cerebellar development by influencing dendrite and synapse formation [56].

Concerning BDNF, its activation and secretion have been shown to be regulated by post-transcriptional mechanisms driven by Shh signaling [14–16]. Furthermore, our previous studies demonstrated reduced availability and reception of Shh signal in *Npc1* mice during the first weeks of cerebellar development [10, 11], which could explain the reduced levels of BDNF in the developing cerebellum, observed in our mouse model. Moreover, a reduction in BDNF expression during embryonic and early postnatal development was also found in the cerebral cortex, brainstem, and cerebellum of mouse models of Gaucher disease, another autosomal recessive genetic disorder of lysosomal storage function [57].

It is well established that BDNF and its TrkB receptor represent critical determinants of cerebellar functions [22, 58]. BDNF, in particular, is mainly present in the GC and PC layers [59, 60] and its expression increases during the maturation of GCs [61–63].

In *Npc1*^*nmf164*^ mice, by immunohistochemistry experiments on cerebellar slices, we also showed a reduced intensity of BDNF mainly at PN11; this result is consistent with the reduced proliferation of GCP deficit in the EGL [10]. As development proceeds (PN15, 30), the reduction of the BDNF protein in the PCs of mutant mice and its concomitant increase in the MF axon terminals become very clear. Since PCs receive BDNF from GCs [59, 64], *Npc1*-related BDNF deficiency in PCs can be directly attributed to the reduced number of GCs; meanwhile, increased BDNF in MFs suggests an autocrine compensating mechanism for the recovery of BDNF signaling. Interestingly, unchanged levels of BDNF protein were detected in young adult mutant mice compared to *wt* mice. We believe that this result reflects the relationship between BDNF levels and the progressive physiological accumulation of gangliosides in our mouse model, starting at PN15 [29]. Indeed, it is known that the accumulation of gangliosides and cholesterol triggers the synthesis and release of mature BDNF [65, 66], which in turn reduces the uptake/internalization of cholesterol in neurons, a neuroprotective mechanism to limit the harmful accumulation of this sterol [67, 68].

To understand whether BDNF downstream signaling was also altered, we evaluated the expression levels of the active form of the TrkB receptor (pTrkB) at different stages of postnatal cerebellar development. Although we demonstrated decreased levels of BDNF at PN8, PN11, and PN15, with respect to its receptor pTrkB, we found significant expression downregulation only at PN8 and PN11 but not at PN15, both in cerebella and purified GCs of *Npc1*^*nmf164*^ mice, compared to *wt* mice. No significant difference was found in the cerebellar expression of BDNF and pTrkB between *Npc1*^*nmf164*^ and *wt* mice at PN30.

Even in BDNF-KO mice, a 50% reduction in BDNF mRNA and protein levels strongly downregulates TrkB receptor activation in the early stages of postnatal development while showing normal pTrkB protein levels in adulthood [69, 70]. Indeed, during postnatal development, BDNF-induced TrkB activation decreases with a predominance of BDNF-independent TrkB activation mechanisms in later stages [71, 72]. This mechanism is known as “transactivation” and involves the activation of G protein-coupled receptors (GPRs) and the binding of other neurotrophins, such as NT3/NT4 to the receptors [72]. Furthermore, a perturbation of TrkB activation is also present in various animal models of ciliopathies [73, 74]. The reduction of pTrkB observed in our model could therefore be a direct consequence of the shortening of the length of primary cilium together with a decrease in the ciliated cell fraction observed in the striatum and cerebellum of *Npc1* mutant mice and in human fibroblasts derived from NPCD patients [11, 74].

BDNF also acts as the main “directional” signal for the migration of GCPs. Indeed, GCPs migrate along a BDNF gradient from EGL to IGL which stimulates TrkB endocytosis, causing asymmetric accumulation of signaling endosomes in the subcellular region where BDNF concentration is highest (polarized GCs) [22]. Therefore, BDNF exocytosis and TrkB endocytosis allow the precursors to polarize and migrate to IGL correctly [22].

By immunofluorescence analysis on PN8 cerebellar sections and DIV3 GCs, we demonstrated that the localization of pTrkB exhibits a homogeneous distribution pattern in the soma of GCs of *Npc1* mutant mice, in contrast to the “polarized” receptor localization seen in *wt* neurons. Furthermore, *Npc1*^*nmf164*^ mice exhibit reduced receptor localization in early endosomes and minor fractions of neurons with BDNF-dependent pTrkB polarization, both in vivo and in vitro GCs. Similar distribution patterns have been described in GC of BDNF-KO mice [22]. In agreement with the disturbance of pTrkB subcellular localization, the response of GCs to exogenous BDNF determined by chemotaxis assay on GCs isolated from PN6 mice, GCs isolated from mutant mice displayed a reduced reactivity to exogenous BDNF compared to *wt.* It is interesting to note that our results are supported by previous in vitro studies that observed reduced responsiveness to BDNF in embryonic striatal neurons isolated from *Npc1* mice, in which cholesterol metabolism is impaired [17].

Using Golgi-Cox staining of the cerebellar sections at PN30, when the GCs in the IGL have completed their differentiation, we also demonstrated defective differentiation of the GCs in the *Npc1* mutant mice. In fact, these mice had dendritic anomalies, with an increase in the length, diameter, and number of branches and dendritic nodes. This phenotype might reflect the additional role of BDNF not only in promoting GC migration but also in inducing axonal and dendritic arborization.

Finally, considering the cerebellar glomerular rosette, a multisynaptic area with two different axonal (mossy and Golgi) and dendritic (granule and Golgi) terminations [48, 75], the cerebella of *Npc1*^*nmf164*^ mice at PN11 and PN30 showed a reduced number of synaptophysin-positive glomeruli, which were also less convoluted and smaller. In addition, from this analysis, it emerged that in *Npc1*^*nmf164*^ mice there is a significant increase in the distance between GCs, probably due to the already demonstrated reduced number of these neurons [10] and a reduction in the GC-glomerulus distance, present only at the stage of PN11.

These findings suggest that in *Npc1* mutant mice, during cerebellar maturation, fewer GCs accumulate around the glomeruli and that the lack of GC-secreted factors, such as BDNF, generates defective morpho-functional maturation of MF-GC synapses in mice [49, 55]. Importantly, MFs are involved in the saccadic movement of the eyes [76] and the anomalies in the maturation of MF-GC synapses demonstrated here could contribute to vertical gaze supranuclear palsy (VSGP) which, together with cerebellar ataxia, is the main clinical sign of human NPC1 pathology [77]. Finally, these synaptic anomalies align with previous evidence of a general imbalance of glutamatergic/GABAergic stimulation that PCs receive from climbing/parallel fibers and basket/stellate cells, respectively [5, 13].

Together, these data indicate that abnormal expression of BDNF is part of complex cerebellar deficits, which may be responsible for subsequent symptomatic events associated with NPC1 pathology. Indeed, an altered pattern of synaptic inputs to PCs can affect the timing of their maturation/activation and ultimately lead to behavioral abnormalities. We believe that the selective vulnerability of these cells and the ataxic phenotype observed in *Npc1* adult mice and patients represent the “final” result of a series of “silent” events of defective cerebellar neurodevelopment.

## Supplementary Information

Figure 1 SM: There was no significant difference observed at PN4 between the levels of BDNF protein in *wt* and *Npc1*^*nmf164*^ mice. The figure presents a representative immunoblot showing the expression levels of cerebellar BDNF protein in *wt* (empty bars) and *Npc1*^*nmf16*^ (full bars) mice at PN4. The BDNF protein levels were normalized to the expression levels of the housekeeping protein, β-actin. The histograms indicate the abundance of BDNF protein expression as mean ± SD (4 mice per group). (PDF 524 kb)

Figure 2 SM. *Npc1*^*nmf164*^ mice display abnormal mossy fibers. Representative fields of parasagittal sections of *wt* and *Npc1*^*nmf164*^ mouse cerebella are shown in the figure. Detection of BDNF (green) and nuclei (Hoechst 33258, blue) by immunofluorescence in PN30 cerebellar sections of *wt* and *Npc1*^*nmf164*^ mice. EGL: External Granular Layer; ML: Molecular Layer; PCL: Purkinje Cell Layer; IGL: Internal Granular Layer; asterisks point mossy fibers; white arrowheads point PCs. scale bars: 20 μm. (PDF 1195 kb)

Figure 3 SM. Absence of cholesterol accumulation in DIV7 CGs derived from *wt* and *Npc1*^*nmf164*^ mice. The presence of unesterified cholesterol was examined using Filipin staining (shown in white) in GCs cultured in vitro for 7 days (DIV). Nuclei were counterstained with propidium iodide (red). (PDF 465 kb)

Figure 4 SM. Staining of Glomeruli in *c*erebellar sections of *wt* and *Npc1*^*nmf164*^mice. Glomeruli were detected using an alternative molecular marker, the vesicular glutamate transporter (Vglut1) (brown) [7, 25], in *wt* and *Npc1* mice at PN30. ML: Molecular Layer; PC: Purkinje Cell; IGL: Internal Granular Layer; Arrowheads = glomeruli. scale bars: 40 μm. (PDF 3412 kb)

Table 1 SM: Quantitative analysis of BDNF histological and staining along cerebellar cortex at different stages of development. ImageJ quantification of the area covered by BDNF staining at PN11, 15, 30 and 90 in wt and Npc1nmf164 mice in four different regions of the cerebellum; n=3. Data are media ± S.E.M (Unpaired t-test). EGL= External Granular Layer; ML: Molecular Layer; PCL= Purkinje Cell Layer; IGL= Internal Granular Layer; WM: White Matter. (PDF 272 kb)
