# Supplementary figures and images for: Reduced Cerebellar BDNF Availability Affects Postnatal Differentiation and Maturation of Granule Cells in a Mouse Model of Cholesterol Dyshomeostasis
Source: Mol Neurobiol. 2023 Jun 14;60(9):5395–410. doi: 10.1007/s12035-023-03435-3 (PMC10415459; doi:10.1007/s12035-023-03435-3)

Figure 1 SM

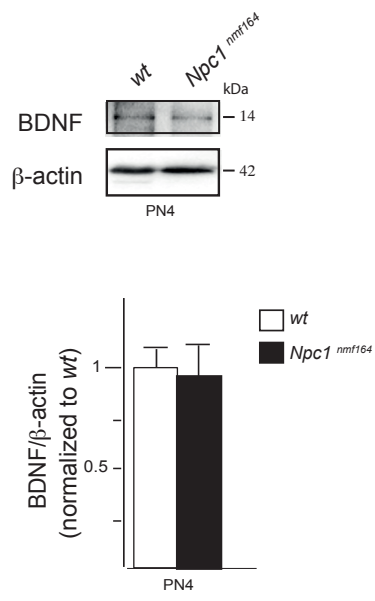

Supplement: Supplementary file 1 — SM: There was no significant difference observed at PN4 between the levels of BDNF protein in wt and Npc1nmf164 mice. The figure presents a representative immunoblot showing the expression levels of cerebellar BDNF protein in wt (empty bars) and Npc1nmf16 (full bars) mice at PN4. The BDNF protein levels were normalized to the expression levels of the housekeeping protein, β-actin. The histograms indicate the abundance of BDNF protein expression as mean ± SD (4 mice per group). (PDF 524 kb) [file 12035_2023_3435_MOESM1_ESM.pdf]

Figure 2 SM

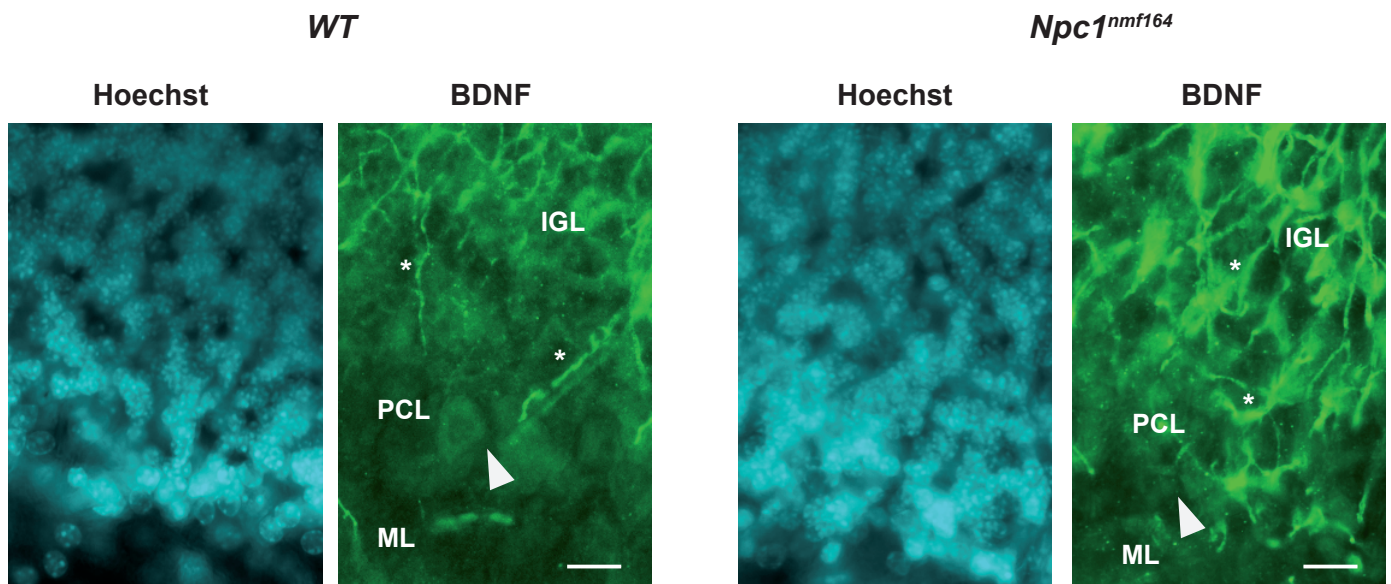

Supplement: Supplementary file 2 — SM. Npc1nmf164 mice display abnormal mossy fibers. Representative fields of parasagittal sections of wt and Npc1nmf164 mouse cerebella are shown in the figure. Detection of BDNF (green) and nuclei (Hoechst 33258, blue) by immunofluorescence in PN30 cerebellar sections of wt and Npc1nmf164 mice. EGL: External Granular Layer; ML: Molecular Layer; PCL: Purkinje Cell Layer; IGL: Internal Granular Layer; asterisks point mossy fibers; white arrowheads point PCs. scale bars: 20 μm. (PDF 1195 kb) [file 12035_2023_3435_MOESM2_ESM.pdf]

Figure 3 SM

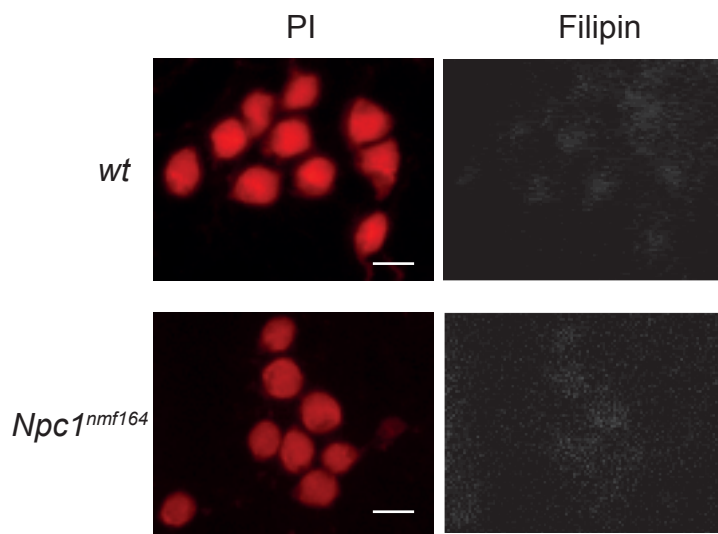

Supplement: Supplementary file 3 — SM. Absence of cholesterol accumulation in DIV7 CGs derived from wt and Npc1nmf164 mice. The presence of unesterified cholesterol was examined using Filipin staining (shown in white) in GCs cultured in vitro for 7 days (DIV). Nuclei were counterstained with propidium iodide (red). (PDF 465 kb) [file 12035_2023_3435_MOESM3_ESM.pdf]

Figure 4 SM

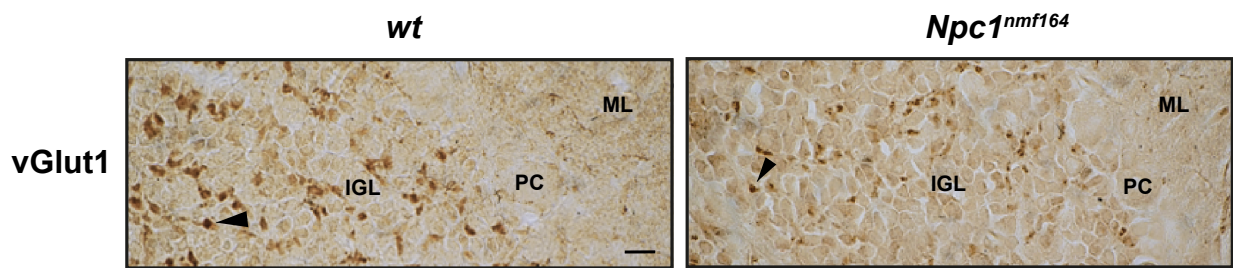

Supplement: Supplementary file 4 — SM. Staining of Glomeruli in cerebellar sections of wt and Npc1nmf164mice. Glomeruli were detected using an alternative molecular marker, the vesicular glutamate transporter (Vglut1) (brown) [7, 25], in wt and Npc1 mice at PN30. ML: Molecular Layer; PC: Purkinje Cell; IGL: Internal Granular Layer; Arrowheads = glomeruli. scale bars: 40 μm. (PDF 3412 kb) [file 12035_2023_3435_MOESM4_ESM.pdf]
