# Supplementary material for: Reduced Cerebellar BDNF Availability Affects Postnatal Differentiation and Maturation of Granule Cells in a Mouse Model of Cholesterol Dyshomeostasis
Source: Mol Neurobiol. 2023 Jun 14;60(9):5395–410. doi: 10.1007/s12035-023-03435-3 (PMC10415459; doi:10.1007/s12035-023-03435-3)
Supplement: Supplementary file 5 — SM: Quantitative analysis of BDNF histological and staining along cerebellar cortex at different stages of development. ImageJ quantification of the area covered by BDNF staining at PN11, 15, 30 and 90 in wt and Npc1nmf164 mice in four different regions of the cerebellum; n=3. Data are media ± S.E.M (Unpaired t-test). EGL= External Granular Layer; ML: Molecular Layer; PCL= Purkinje Cell Layer; IGL= Internal Granular Layer; WM: White Matter. (PDF 272 kb) [file 12035_2023_3435_MOESM5_ESM.pdf]

Age	Area	Intensity (mean)		P value
		wt	Npc1 ^{nmf164}	
PN11	EGL	36053	19751	0,0110
	PCL	83940	28661	<0,0001
	ML	214393	149402	0,0429
	IGL	91248	58241	0,0236
	WM	192771	121923	0,0310
PN15	ML	81132	16806	0,0178
	PCL	77659	39329	0,0236
	IGL	49425	118611	0,0164
PN30	PCL	124459	16674	0,0014
	IGL	65953	111542	0,0362
PN90	IGL	178466	157062	NS

Table 1 SM: Quantitative analysis of BDNF histological and staining along cerebellar cortex at different stages of development. *ImageJ quantification of the area covered by BDNF staining at PN11, 15, 30 and 90 in wt and Npc1*^{nmf164} *mice in four different regions of the cerebellum; n=3. Data are media ± S.E.M (Unpaired t-test). EGL= External Granular Layer; ML: Molecular Layer; PCL= Purkinje Cell Layer; IGL= Internal Granular Layer; WM: White Matter.*
